# Predicting effective pro-apoptotic anti-leukaemic drug combinations using co-operative dynamic BH3 profiling

**DOI:** 10.1371/journal.pone.0190682

**Published:** 2018-01-03

**Authors:** Martin Grundy, Claire Seedhouse, Thomas Jones, Liban Elmi, Michael Hall, Adam Graham, Nigel Russell, Monica Pallis

**Affiliations:** 1 Clinical Haematology, Nottingham University Hospitals, Nottingham, United Kingdom; 2 Department of Haematology, Division of Cancer and Stem Cells, University of Nottingham, Nottingham, United Kingdom; University of Illinois at Chicago, UNITED STATES

## Abstract

The BH3-only apoptosis agonists BAD and NOXA target BCL-2 and MCL-1 respectively and co-operate to induce apoptosis. On this basis, therapeutic drugs targeting BCL-2 and MCL-1 might have enhanced activity if used in combination. We identified anti-leukaemic drugs sensitising to BCL-2 antagonism and drugs sensitising to MCL-1 antagonism using the technique of dynamic BH3 profiling, whereby cells were primed with drugs to discover whether this would elicit mitochondrial outer membrane permeabilisation in response to BCL-2-targeting BAD-BH3 peptide or MCL-1-targeting MS1-BH3 peptide. We found that a broad range of anti-leukaemic agents–notably MCL-1 inhibitors, DNA damaging agents and FLT3 inhibitors–sensitise leukaemia cells to BAD-BH3. We further analysed the BCL-2 inhibitors ABT-199 and JQ1, the MCL-1 inhibitors pladienolide B and torin1, the FLT3 inhibitor AC220 and the DNA double-strand break inducer etoposide to correlate priming responses with co-operative induction of apoptosis. ABT-199 in combination with pladienolide B, torin1, etoposide or AC220 strongly induced apoptosis within 4 hours, but the MCL-1 inhibitors did not co-operate with etoposide or AC220. In keeping with the long half-life of BCL-2, the BET domain inhibitor JQ1 was found to downregulate BCL-2 and to prime cells to respond to MS1-BH3 at 48, but not at 4 hours: prolonged priming with JQ1 was then shown to induce rapid cytochrome C release when pladienolide B, torin1, etoposide or AC220 were added. In conclusion, dynamic BH3 profiling is a useful mechanism-based tool for understanding and predicting co-operative lethality between drugs sensitising to BCL-2 antagonism and drugs sensitising to MCL-1 antagonism. A plethora of agents sensitised cells to BAD-BH3-mediated mitochondrial outer membrane permeabilisation in the dynamic BH3 profiling assay and this was associated with effective co-operation with the BCL-2 inhibitory compounds ABT-199 or JQ1.

## Background

The modes of action of diverse cytotoxic agents generally converge on mitochondrial apoptotic pathways [1]. To allow apoptosis to occur, effector molecules BAX and BAK must oligomerise to form pores that cause mitochondrial outer membrane permeabilisation (MOMP). BAX and BAK activation can be triggered by BH3-only proapoptotic BCL-2 family members such as BID and BIM, PUMA, BAD and NOXA. These are opposed by BCL-2 family prosurvival members, such as MCL-1 and BCL-2 itself, that sequester pro-apoptotic family members to hold apoptosis in check. Effective pro-apoptotic drugs alter the equilibrium of the system, both by altering relative levels of the pro-and anti-apoptotic BCL-2 family members and triggering changes of phosphorylation, conformation and location [1, 2].

Monotherapies are not successful at inducing remissions in patients with acute myeloid leukaemia. With many new drugs on the market or in the pipeline [3, 4], there is a need to establish rational principles for predicting suitable drug combinations. One such principle is co-operation between agents that activate complementary components of pro-apoptotic pathways. For example, the sensitiser molecule BAD is ineffective against MCL-1, and NOXA is ineffective against BCL-2, but there is direct co-operation between BAD and NOXA in mediating apoptosis [5], suggesting that therapeutic agents that inhibit BCL-2 may complement agents that inhibit MCL-1. Indeed, several studies have now shown synergy between specific BCL-2 and MCL-1 antagonists [6–10]. Mechanistically, when BCL-2 is inhibited, e.g. by the binding agents ABT-737 or ABT-199, the apoptosis activator BIM is released [7, 10–13], but the released BIM can then be taken up by MCL-1, so protection from apoptosis is maintained unless MCL-1 is also antagonised [10, 14–16].

There is currently great interest in discovering the ability of different classes of therapeutic agents to synergise with BCL-2 or MCL-1 antagonists [8, 10, 14–23]. As MCL-1 has a short half-life (approximately 1 hour) [24] it can be rapidly downregulated, as reported after treatment with ultraviolet radiation [25] or sorafenib [26] for 3 hours or less. It is unclear whether this is crucial for induction of apoptosis, since other factors, such as induction of BIM, NOXA or PUMA are also reported. BCL-2 is a much more stable protein than MCL-1, with a protein half-life of approximately 14 hours [27, 28]. BCL-2 downregulation can be effected by BET domain inhibitors, but whereas message downregulation occurs rapidly in sensitive cells, protein loss takes place over a much longer time period [29]. In contrast, BCL-2 binding antagonists such as ABT-737 or ABT-199 (venetoclax) [30, 31] can act rapidly to induce apoptosis in sensitive cells.

Whilst several authors have documented the efficacy of the BCL-2 antagonists ABT-199 and ABT-737 at co-operating with agents that downregulate or bind MCL-1 [6, 8, 10, 16–19, 21, 22], the literature is focused on individual drug combinations. In the current study we use a variety of drugs and chemical inhibitors to systematically identify agents sensitising to BCL-2 antagonism and agents sensitising to MCL-1 antagonism. Dynamic BH3 profiling [32] is a novel methodology that measures the capacity of drugs to prime mitochondria for apoptosis, and involves the addition of permeable pro-apoptotic BCL-2 family BH3 peptides to drug primed cells to induce speedy mitochondrial outer membrane permeabilisation (MOMP). In this study we measure MOMP after applying BAD (BCL-2 targeting) or MS1 (MCL-1 targeting) BH3 peptides to the drug-primed cells. Induction of MOMP is measured here with a cytochrome C release assay [33]. Applying this technique previously, we had shown that the MCL1 downregulator TG02 sensitises to the BCL-2-inhibitory BAD-BH3 peptide, whereas the BCL-2 antagonist ABT-199 sensitises to MCL-1 inhibitory NOXA-BH3 peptide, and the two agents synergise in dual-sensitive cells to induce apoptosis [18]. We dichotomise drugs as either agents sensitising to BCL-2 antagonism or agents sensitising to MCL-1 antagonism, and we demonstrate the efficacy of combining an agent from each category in apoptosis assays.

## Materials and methods

### Materials

Drugs and suppliers used in the study were as follows: 17-AAG, rapamycin, sorafenib and torin1 from LC labs (www.lclabs.com); AC220, JQ1, selinexor, tosedostat, TW-37 and vosaroxin from Selleck (supplied by Stratech UK); ABT-199 from Adooq, www.adooq.com; ABT-737 from Sequoia, Pangbourne, UK; A-1210477 from Chemie Tek, www.chemietek.com; etoposide from Tocris, Bristol, UK; Mylotarg from Wyeth, Pearl River USA; Pladienolide B from Santa Cruz, supplied by Insight, Wembley, UK; TG02 was from Tragara, San Diego, USA. Other drugs and reagents were from Sigma (Poole, Dorset, UK) unless specified.

### Cells

The MV4.11 cell line was from the American Tissue Culture Collection (Manassas, USA) and was maintained in RPMI 1640 medium with 10% foetal calf serum (FCS; First Link, Birmingham, UK), and 2mM L-glutamine. All cultures were kept at 37°C in 5% CO_2_ and all experiments were performed with cell lines in log phase. Continued testing to authenticate these cell lines was performed using multiplex short tandem repeat analysis (Powerplex 16, Promega, Southampton, UK). Mycoplasma testing was carried out routinely using the Mycoalert mycoplasma detection kit (Lonza, Rockland, USA) and following the manufacturer’s instructions.

### Dynamic BH3 profiling

MV4.11 cells were incubated (5 X 10^5^/ml) in RPMI with 10% FCS for four hours with the indicated drugs. Cytochrome C release (using Alexa-647-conjugated cytochrome C antibody, BD #558709) was measured by flow cytometry after a further 60 minute incubation of digitonin-permeabilised cells with BH3 peptides as described [18, 33]. Adjustments for peptide induced cytochrome C release in untreated cells were made in order to establish agent-specific release (delta priming), using the formula (percent release with agent and peptide–percent release with peptide)/ (100 –percent release with peptide). A mutated PUMA-BH3 peptide (PUMA2A) [33] was used at 100 μM as control in all experiments.

### Determination of apoptosis

Cytochrome C release, loss of mitochondrial membrane potential (↓Δψ_m_) and uptake of 7-amino-actinomycin D were measured by flow cytometry.

To measure the percentage of cells with loss of Cytochrome C, cells were fixed in 2% paraformaldehyde directly after a 4 hour drug incubation. Fixed and rinsed cells were permeabilised with saponin and incubated overnight with Alexa-647-conjugated cytochrome C antibody and analysed by flow cytometry.

The percentage of cells with loss of mitochondrial membrane potential (↓Δψ_m_) was determined following incubation of cells as previously reported [34] with the fluorescent dye 3,3’–dihexyloxacarbocyanine iodide (DiOC_6_, 40 nM, Molecular Probes, Oregon) for the final 75 minutes of a 5 ¼ hour drug incubation, with 0.5 μg/ml 7-aminoactinomycin D (7-AAD) also added for the final 30 minutes to measure a later stage of apoptosis [35].

### Protein measurement

Protein expression of 4E-BP1 p-thr36/45, BD#560286) and BCL-2 (Ancell #357–040) were measured by flow cytometry. MCL-1L (long) and MCL-1S (short) forms were measured by Western Blotting using the sc-819 antibody from Santa Cruz.

### MCL1 mRNA short and long form measurement

Expression levels of Mcl-1L and Mcl-1S using cDNA from AML cell lines were determined quantitatively by qRT-PCR on an ABI Prism 7500 (Applied Biosystems) using SYBR Green Master Mix (Applied Biosystems). Specific amplification of the isoforms was achieved using primers with previously reported sequences [36]. Gene expression levels were normalized to beta-2-microglobulin (β2m) housekeeping gene expression [37]. Negative controls (no template) were included in all the experiments and the reactions were run in triplicate.

### Calculations and statistics

Fold excess addititivism was calculated as a ratio of observed to expected values for drug combinations, where the expected value C is calculated from the Bliss algorithm for response to two compounds with effects A and B i.e. C = A + B–A*B [38, 39]. This method allows for potentiation and augmentation as well as synergism. Statistics were carried out using SPSS version 22 software (Chicago, IL, USA). P values <0.05 were considered statistically significant.

## Results

### 1. Methodology

**Fig 1:**
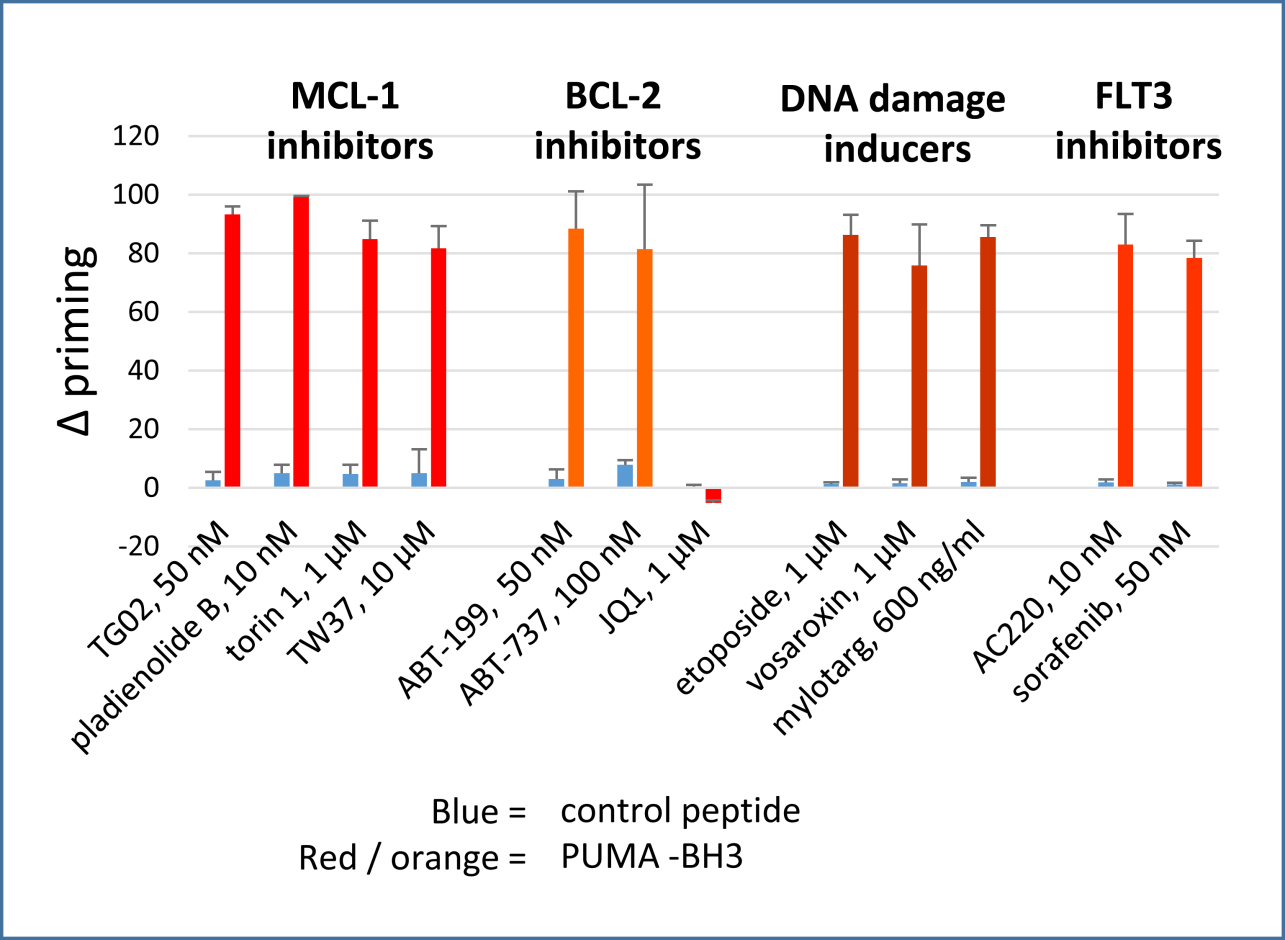
Dynamic BH3 profiling assay: delta priming to PUMA-BH3. The increase in cells primed to undergo mitochondrial outer membrane depolarisation (Δ priming) is measured by Cytochrome C release after 4 hour drug treatment. PUMA-BH3 was used at 3 μM. Values are corrected for Cytochrome C release with peptide only as described in the methods. (Mean+/- SD for n = 3).

MV4-11 cells were used for screening drugs, as these cells were highly sensitive to apoptosis induced by either BCL-2 or MCL-1 targeting (S1 Fig). These cells do not over-express BCL-X_L_ [18]. Montero and colleagues [32] have described the technique of dynamic BH3 profiling, which involves using short drug exposures to prime mitochondria for BH3 peptide-induced Cytochrome C release, and these authors demonstrated that the assay could predict cytotoxicity. Dynamic BH3 profiling relies on the ability of a drug and a BH3-only pro-apoptotic peptide to induce MOMP in cells when used in combination: sensitivity is compromised if the agent or peptide induce too much apoptosis individually, so it is crucial to establish suitable drug concentrations for the assay. To allow drugs to prime cells as single agents, but not to kill them outright during the course of the assay, we established a suitable incubation time and drug concentrations by screening with a PUMA-BH3 peptide which can sensitise all the anti-apoptotic BCL-2 family proteins [28]. We defined appropriate priming concentrations of agents as those that induced >75% Cytochrome C release in the presence of PUMA-BH3 but less than 10% when incubated with a control peptide, i.e. a >65% change in priming (“Δ priming”, Fig 1). The data show that a variety of agents prime to PUMA-BH3 after as little as four hours. Agents used in the study along with their proposed mechanism of sensitising to apoptosis are delineated in Table 1. We grouped agents into four main categories. The first category comprised agents expected to strongly inhibit MCL-1. The second category of agents comprised BCL-2 antagonists. Agents which induce double strand breaks (etoposide, mylotarg, and vosaroxin—category 3) and FLT3 inhibitors (AC220, sorafenib—category 4) were also studied. All the agents studied primed to PUMA-BH3 after a four-hour incubation with the exception of JQ1. Rationales and results for priming to PUMA-BH3 for several additional agents (miscellaneous, category 5) are shown in S2 Fig.

**Table 1:** Agents expected to antagonise or downregulate BCL-2 or MCL-1.

	Agent	Expected or potential mechanism	Rapid (within 4 hours) or delayed response
**1. MCL-1 inhibitors**			
	Pladienolide B	Alternative splicing of MCL-1[40]	MCL-1 protein decrease at 4 hours (current study)
	TG02	Protein synthesis inhibition downregulating MCL-1 [18, 41]	MCL-1 protein decrease at 4 hours
	Torin1	Protein synthesis inhibition [19, 42]	MCL-1 protein decrease at 4 hours (current study)
	TW-37	Direct inhibition of BCL-2 and MCL-1 in cell free system [43]	
		Preference for MCL-1 in cellular systems [44]	Early timepoints not tested
**2. BCL-2 inhibitors**			
	ABT-199	Direct BCL-2 binding and antagonism [31]	Apoptosis within 4 hours
		Release of BIM/BAX from BCL-2 [15, 45]	rapid
	ABT-737	Direct BCL-2 binding and antagonism [30]	timings not reported
		Release of BIM/BAX from BCL-2 [11, 12]	rapid
	JQ1	Downregulation of BCL-2 [29, 46]	Rapid decrease in message. Slow decrease in protein
**3. DNA damaging agents**			
	etoposide	Downregulation of MCL-1 [47]	Within 8 hours. Earlier time points not studied
		Activation of ceramide [48, 49] (associated with BCL-2 inhibition and BAD activation [50, 51]	Rapid
	mylotarg	Possibly as reported for etoposide	
	vosaroxin	Possibly as reported for etoposide	
**4. FLT3 inhibitors**			
	sorafenib	Downregulation of MCL-1 [17, 26]	rapid
		Inactivation of ERK associated with bcl-2 dephosphorylation [26]	rapid
	AC220	Possibly as reported for sorafenib	

We previously showed that the MCL-1 reducing agent TG02 primes AML cells to respond to BCL2 antagonism and that ABT-199 primes cells to respond to MCL-1 antagonism [18]. In that assay a NOXA-BH3 peptide was used to antagonise MCL-1. However we refined the methodology for the current study following reports that the MS1 peptide binds to MCL-1 with higher affinity than NOXA-BH3 [52]. In MV4.11 cells the concentration of MS-1 required to complement ABT-199 was 10-100-fold less than that of NOXA-BH3 (S3 Fig). On the basis of this data we selected 3 μM BAD-BH3 and 3 μM MS1-BH3 for the complementary profiling assay.

### 2. Co-operative dynamic BH3 profiling

**Fig 2:**
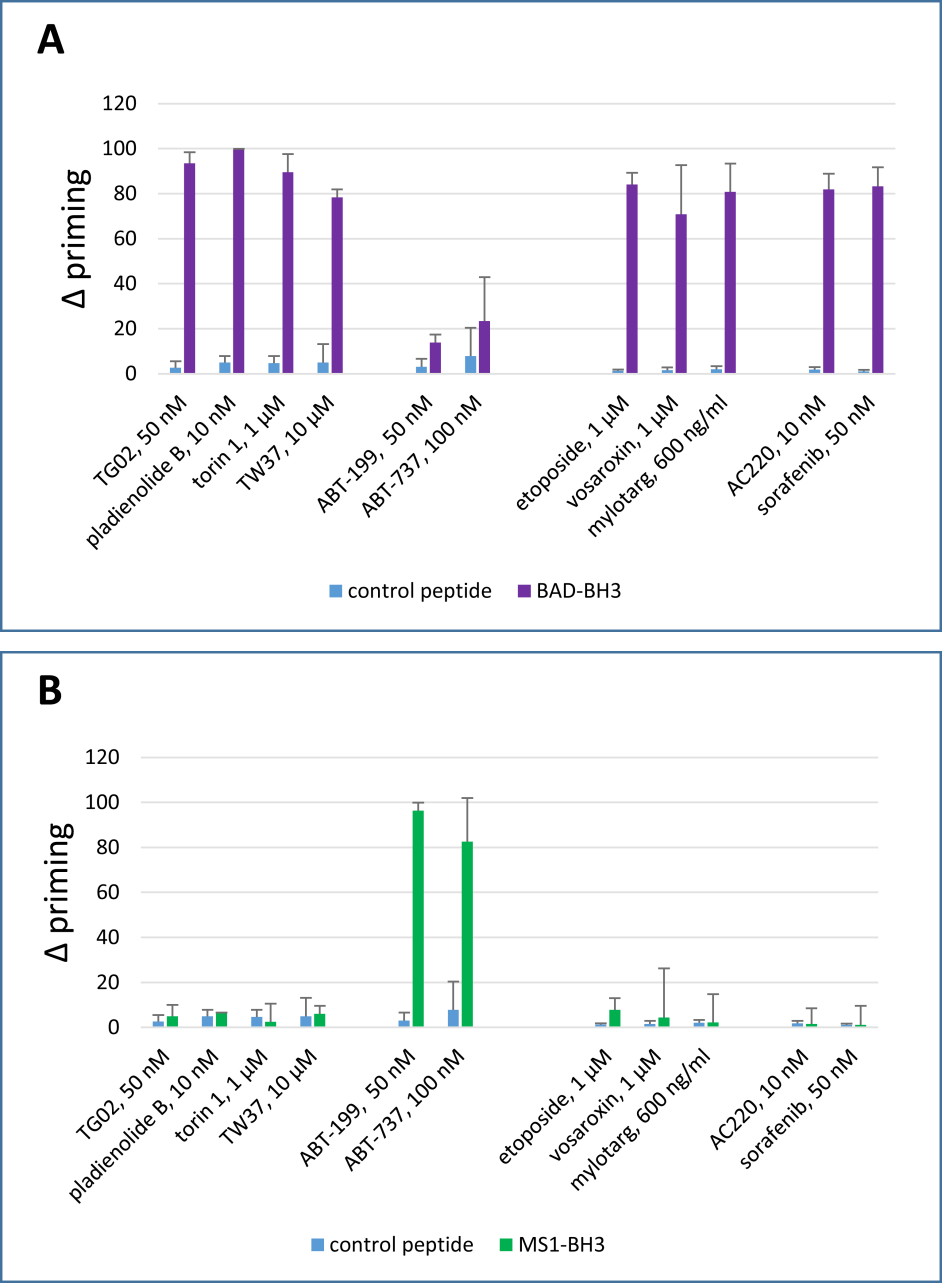
**Dynamic BH3 profiling assay: delta priming to (A) BAD-BH3 and (B) MS1-BH3 peptides.** Delta priming is measured by cytochrome C release after 4 hour drug treatment and additional incubation with the indicated BH3 peptides (BAD-BH3 at 3 μM, MS1-BH3 at 3 μM, PUMA2A control at 100 μM). Values are corrected for cytochrome C release with peptide only as described in the methods. (Mean+/- SD for n = 3).

Cells were incubated for four hours with agents of interest followed by dynamic BH3 profiling. Diverse agents sensitised cells to BAD-BH3, indicating that the four hour drug incubation had increased the BCL-2 dependence of the cells and thus enabled the cells to cross the threshold for apoptosis once the BCL-2 antagonist peptide was added (Fig 2A and S4 Fig). In contrast, only the highly specific BCL-2 antagonist ABT-199 and the BCL-2/BCL-XL antagonist ABT-737 primed to equimolar (3 μM) MS1: i.e. after priming with a variety of drugs, MCL-1 antagonism with MS1-BH3 did not generally enable the cells to cross the threshold for apoptosis (Fig 2B and S4 Fig).

### 3. Co-operative induction of apoptosis

**Fig 3:**
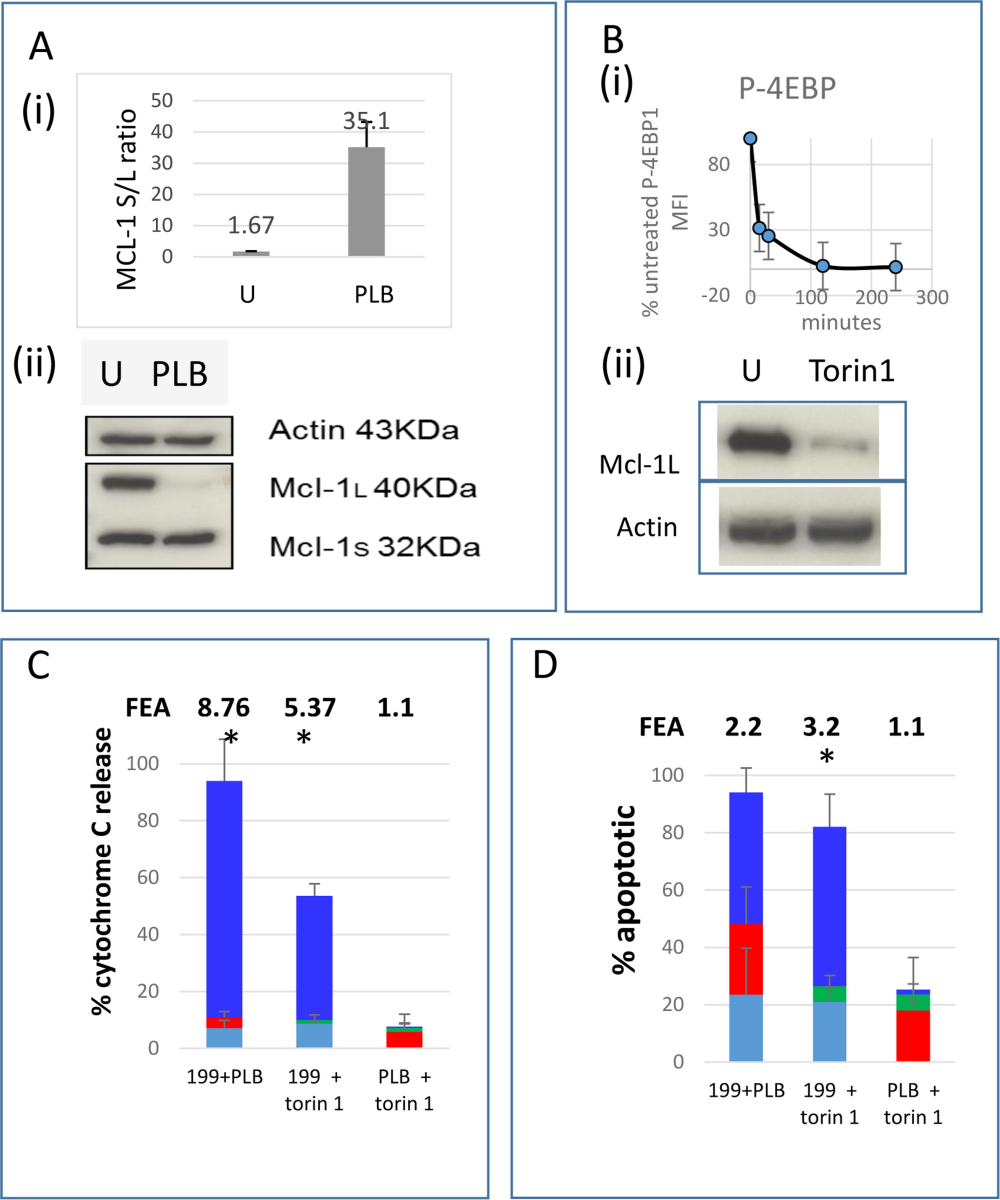
Co-operative induction of apoptosis by ABT-199 with pladienolide B or torin1. **(A)** MCL-1 long form (L) and short form (S) transcripts (i) and protein (ii) were quantified in untreated cells and cells treated with 10 nM pladienolide B (PLB) for 4 hours. **(B)** 4E-BP1 phosphorylation (i) and MCL-1 protein (ii) were quantified in untreated cells and cells treated with 1μM torin1 for 4 hours. (**C, D)** Cells were incubated with 10 nM ABT-199 (199, turquoise), 10 nM pladienolide B (PLB, red), 1 μM torin1 (green) or the indicated combinations (bright blue bar, height = effect with both agents in combination–sum of effects with agents individually). **(C)** After 4 hours cells were fixed and processed for Cytochrome C release. **(D)** After 4 hours DiOC6 was added for a further 75 minutes and 7-amino actinomycin D for the last 30 minutes. (Mean+/- SD for n = 3). Fold excess additivism (FEA) is shown on the figures and was calculated as a ratio of observed to expected values after corrections according to the Bliss algorithm (see methods). Asterisks indicate observed values significantly higher than expected values (P<0.05).

On the basis of the BAD-BH3 and MS1-BH3 profiling, we investigated whether the cells would be sensitive to a combination of BCL-2 and MCL-1 antagonists. ABT-199 is well described as a powerful BCL-2 antagonist at nanomolar concentrations in AML cells [53]. From the data illustrated in Fig 2A, we selected two MCL-1 antagonists: pladienolide B and torin1. The spliceosome inhibitor pladienolide B is reported to rapidly induce alternative splicing of MCL-1 from the anti-apoptotic long form to the pro-apoptotic short form [40]. At 10 nM, pladienolide had primed for >99% sensitisation to BAD-BH3 (Fig 2A). We confirmed the reported alternative splicing mechanism in our system and the rapid loss of MCL1 protein (Fig 3A). We also used the powerful chemical mTORC1 antagonist torin1 [42] to inhibit translation, because translational activation by oncogenic kinases is a widespread phenomenon in AML, via STAT5, PIM, ERK and PI3K pathways, which all act on translation initiation factors. Inhibition of translation depletes MCL-1 protein [54]. We confirmed that torin1 dephosphorylates the translational activator 4E-BP1 and depletes MCL-1 in the MV4.11 cells (Fig 3B). A supra-additive effect of combining ABT-199 with either pladienolide B or torin1 to induce cytochrome C release was documented, but no complementary MOMP or apoptosis was detected when MCL-1 targeting agents were used with each other (Fig 3C and 3D).

**Fig 4:**
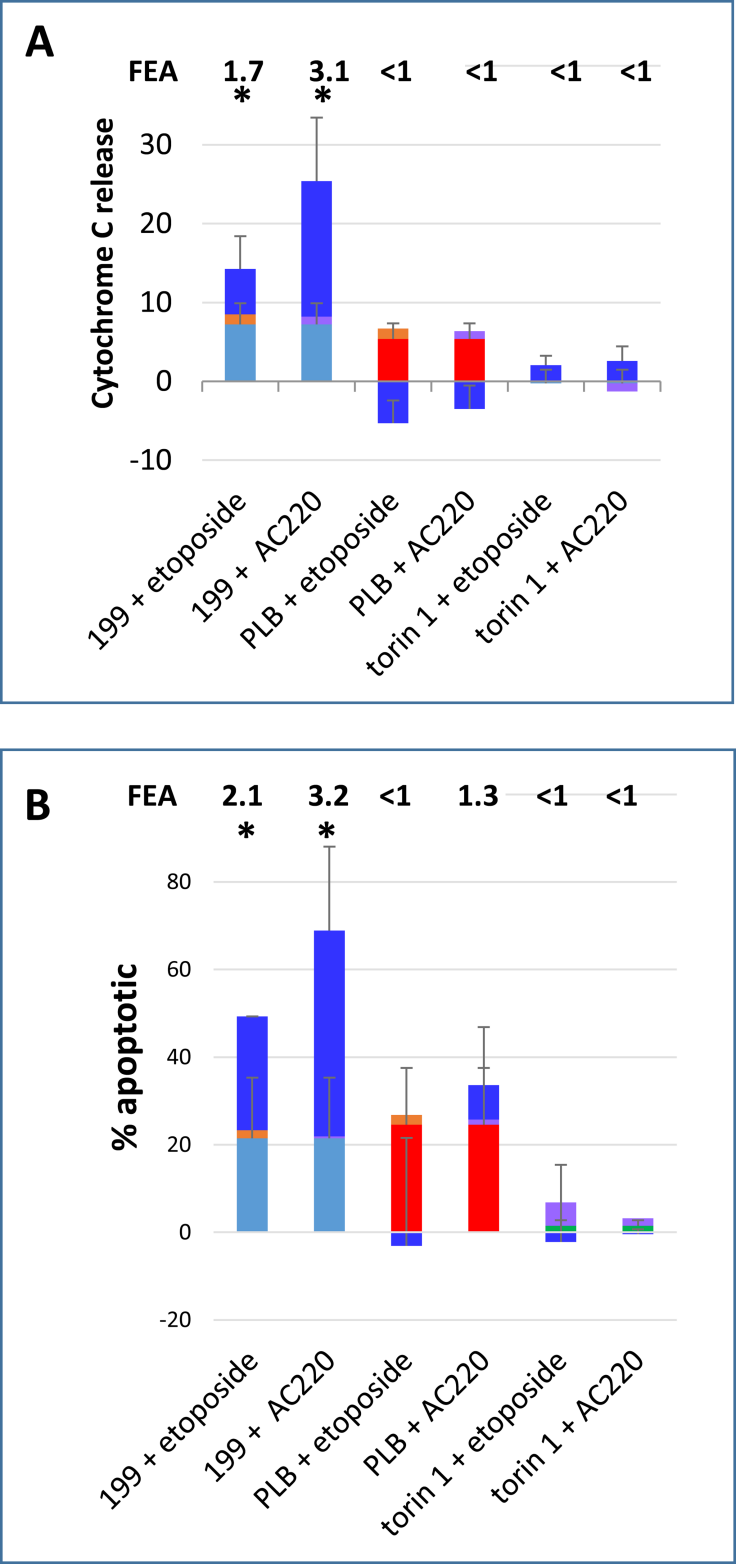
Co-operative induction of apoptosis by ABT-199 with etoposide and AC220. Cells were incubated with 10 nM ABT-199 (199, turquoise), 10 nM pladienolide B (PLB, red), 1 μM torin1 (green), 1 μM etoposide (orange) and 10 nM AC220 (mauve) or the indicated combinations (bright blue bar, height = effect with both agents in combination–sum of effects with agents individually). **(A)** After 4 hours cells were fixed and processed for Cytochrome C release. **(B)** After 4 hours DiOC6 was added for a further 75 minutes and 7-amino actinomycin D for the last 30 minutes. (Mean+/- SD for n = 3). Fold excess additivism (FEA) is shown on the figures and was calculated as a ratio of observed to expected values after corrections according to the Bliss algorithm (see methods). Asterisks indicate observed values significantly higher than expected values (P<0.05).

We expanded the study to include a DNA damage-inducing agent and a FLT3 inhibitor, since these categories of agent are of particular clinical interest in AML and had sensitised to BAD-BH3 in the dynamic BH3 profiling assay. Combining the double strand break-inducing agent etoposide or the FLT3 inhibitor AC220 with ABT-199 led to early MOMP and apoptosis (Fig 4), whereas combining the agents with pladienolide B or torin1 did not have significant pro-apoptotic effects. Flow cytometric illustrations of apoptosis in treated cells are shown in S5 Fig.

### 4. Delayed co-operative induction of apoptosis: JQ1 as a BCL-2 antagonist

**Fig 5:**
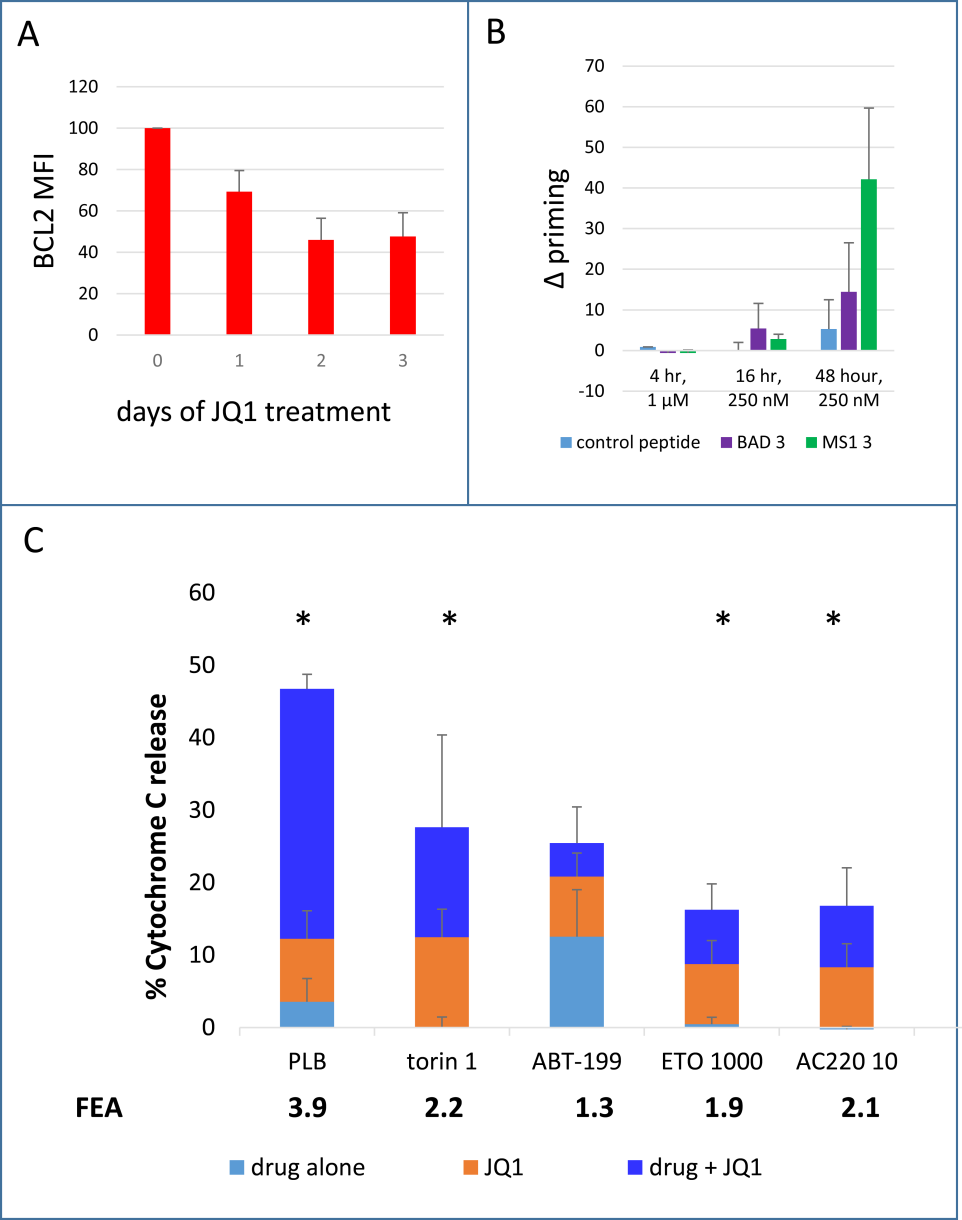
Co-operative induction of apoptosis by pladienolide, torin1, etoposide or AC220 with JQ1. **(A**) Time course of bcl-2 protein downregulation in response to JQ1 measured by flow cytometry. MFI = mean fluorescence intensity, corrected for isotype control. (**B)** Time course of delta priming to BAD-BH3 and MS1-BH3 measured by cytochrome C release after drug treatment and additional incubation with the indicated BH3 peptides. Values are corrected for Cytochrome C release with peptide only as described in the methods. **(C)** Cells were incubated with 250 nM JQ1 for 2 days. 10nM pladienolide B (PLB), 1 μM torin1, 10nM ABT-199 (199), 1 μM etoposide (ETO) or 10 nM AC220 were added for the final 4 hours. Cells were then fixed and processed for Cytochrome C release. Bright blue bar height = cytochrome C release with both agents in combination–sum of cytochrome C release with both agents individually). Fold excess additivism is shown on the figures and was calculated as a ratio of observed to expected values after corrections according to the Bliss algorithm (see methods). Asterisks indicate observed values significantly higher than expected values (P<0.05). (Mean+/- SD for n = 3).

The pattern observed thus far of common MCL-1 antagonism and rare BCL-2 antagonism is likely, at least in part, to be predicated on differences in the stability of the two proteins. MCL-1 has a very short half-life (approximately one hour) [24] which makes it extremely susceptible to rapid downregulation when protein synthesis is decreased or degradation pathways are activated [28, 54]. BCL-2 however, has a much longer half-life (10–14 hours)[27], such that where an agent downregulates BCL-2 at the message level, it may take several hours for functional consequences to be observed. The BET domain inhibitor JQ1 is documented to downregulate BCL-2 [29] and therefore might acquire the capacity to prime for an MS1 (MCL1-antagonist) response. In MV4-11 cells BCL-2 protein downregulation was noted which plateaued at approximately 48 hours, and JQ1 was able to prime to MS-1 BH3 after 48 hours of incubation (Fig 5A and 5B). Cells were therefore incubated with JQ1 for 48 hours with addition of additional agents for the final four hours. JQ1 was found to prime cells to pladienolide, torin1, etoposide and AC220 (Fig 5C). No significant interaction was observed between JQ1 and ABT-199.

### 5. Scheduling-specific dependence of co-operation between ABT-199 and the MCL-1 antagonist A-1210477

**Fig 6:**
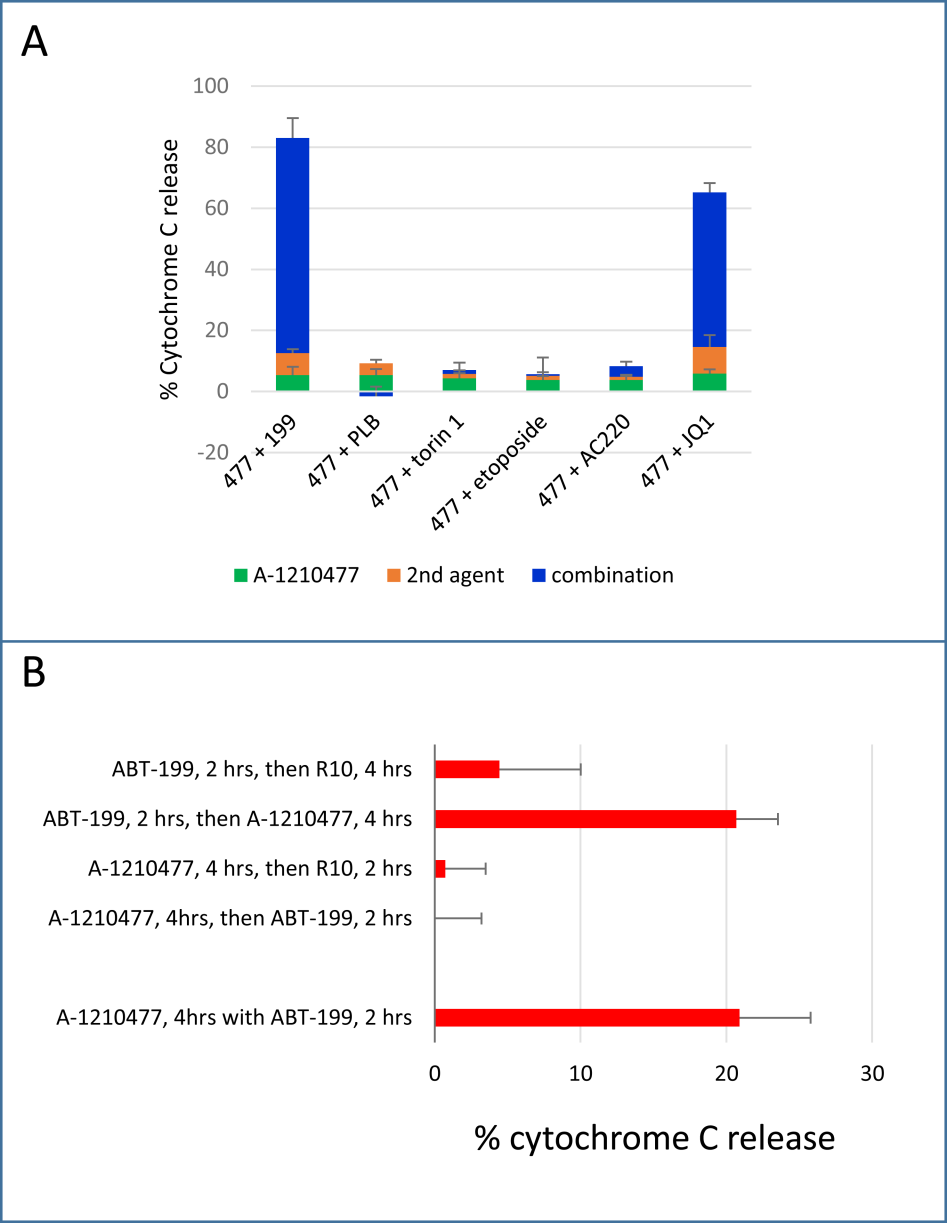
Co-operative induction of apoptosis using A-1210477. A. Cells were co-incubated with 1 μM A-1210477 (477) and with 10 nM ABT-199 (199), 10nM pladienolide B (PLB), 1 μM torin1, 1 μM etoposide or 10 nM AC220 for 4 hours. Alternatively, cells were incubated with JQ1 for 48 hours and A-1210477 was added for the final four hours of the incubation. Cells were then fixed and processed for Cytochrome C release. (Mean+/- SD for n = 3). B. Cells were incubated with 1 μM A-1210477 for four hours and 10 nM ABT-199 was added either before, after or concurrently (final 2 hours). In the two-step conditions, cells were pelleted and rinsed twice in RPMI at 4°C in between agents. R10 = medium without drug.

The specific MCL1 binding inhibitor A-1210477 is an *in vitro* chemical tool that had not been used in our earlier panel as it induced apoptosis in its own right at high concentrations (> 3μM) but failed to prime to BAD-BH3 at sub-micromolar concentrations (S6 Fig). We nevertheless decided to investigate it further because of its specificity for MCL-1 [6]. A supra-additive effect of combining ABT-199 or JQ1 with 1 μM A-1210477 to induce cytochrome C release was documented, but no enhancement of cytochrome C release was detected when A-1210477 was used with the MCL-1 downregulating agents pladienolide B or torin1 or with etoposide or AC220 (Fig 6A), thus providing further evidence of the particular efficacy of combining BCL-2 antagonists with MCL-1 antagonists. As the BH3 profiling assay had not predicted the co-operative potential of 1 μM A-1210477 with BCL-2 antagonists, we investigated this anomaly. A possible explanation is predicated on the fact that, in the BH3 profiling assay, the MCL-1 antagonist A-1210477 and the BCL-2 antagonist (BAD-BH3 peptide) are added to cells sequentially, possibly allowing pro-apoptotic activators such as BIM and PUMA to return to an undepleted pool of MCL-1 when the cells are washed in preparation for addition of peptide in permeabilisation buffer, whereas in the apoptosis experiments A-120477 and ABT-199 are incubated with cells simultaneously. An assay in which we compared sequential with concurrent use of ABT-199 and A-1210477 showed that A-1210477 was indeed ineffective when incubated with cells for four hours and washed off before a two hour incubation with ABT-199 (Fig 6B). However the reverse sequence, i.e. applying ABT-199 before A-1210477, was as effective as using the two agents concurrently.

## Discussion

The technique of co-operative dynamic BH3 profiling has shown that a range of anti-AML drugs and chemical inhibitors sensitise to BCL-2 antagonism and a much smaller number of agents (ABT-199, ABT-737 and JQ1) sensitise to MCL-1 antagonism. MCL-1 and BCL-2 are both guardians of mitochondrial integrity, protecting the healthy cell by sequestering pro-apoptotic BCL-2 family members but allowing controlled release of the pro-apoptotic molecules in response to appropriate stresses. The technique provided evidence that many drugs cause early (within 4 hours) pro-apoptotic changes in cells. Dynamic BH3 profiling does not specify exactly which molecules are effecting these pro-apoptotic changes, but it does show whether these changes can be complemented by specifically antagonising BCL-2 or MCL-1. We add the caveat that A-1210477, and likely other transient binding agents, do not work well in this assay.

Although often described together, there are important functional differences between MCL-1 and BCL-2 which bear upon the findings of the current study. MCL-1 is an unstable protein with a very short half-life [24, 28, 54]. Its rapid destruction may be a common factor in cellular responses to stresses, such that the propensity of major drug types to downregulate MCL-1 may be a dominant cause of sensitising cells to BCL-2 antagonism. Brunelle and colleagues documented that BCL-2 overexpression conferred resistance to a range of chemotherapeutic agents, but the same agents were effective in MCL-1 over-expressing cells. Moreover MCL-1 protein was depleted in sensitive, drug-treated cells [47]. Priming to BCL-2 inhibitors is not exclusively through MCL-1 downregulation: alternative pathways have been documented [55, 56], but a lot of evidence points to MCL-1 downregulation being a very common feature of chemo responsiveness (see Introduction, Table 1 and Fig 3). An inference here might be that MCL-1 binding antagonists become redundant where the drug has caused protein depletion. This could account for the lack of interaction between the antagonist A-1210477 and the spliceosome inhibitor pladienolide or the mTOR inhibitor torin1 in our study, both of which ablated MCL-1. From a different angle, MCL-1 binding antagonists may be useful to supplement the MCL-1 depleting effects of less efficient agents, particularly *in vivo*. Also, the apparent lack of avidity of A-1210477 binding (Fig 6), draws attention to the complication that, whereas transient MCL-1-binding agents may have an advantage in reducing toxicity in a clinical setting, their efficacy in combination with BCL-2 inhibitors may require co-administration.

In contrast to the MCL-1 depleting abilities of many drugs, agents that inhibit BCL-2 are uncommon. ABT-199 has been well characterised in the literature and is being used (as venetoclax) in the clinic. BET domain inhibitors and their interactions with other drugs are less well characterised, but there is currently huge interest in their development [57]. Although BCL-2 downregulation by JQ1 and associated sensitisation to MS1-BH3 were documented in our study, we add the caveat that BET domain inhibitors are highly non-specific and have numerous additional targets [29].

The current set of experiments is applicable to agents that elicit rapid pro-apoptotic changes, which could be a useful strategy to pre-empt protective stress responses. Agents as diverse as etoposide and AC220 sensitised powerfully to BAD-BH3 after just 4 hours (Fig 2). We did not find complementary dynamic BH3 profiling to be useful in the context of nucleoside analogues ara-C or 5-azacytidine, which elicited only weak priming to BAD-BH3 (S4 Fig) or with ABT-199 in 4 hour apoptosis experiments (data not shown). We also did not find the dynamic profiling assay to be useful for predicting synergy in 48 hour dose response assays (data not shown), so the current findings are likely confined to early interactions. The original dynamic BH3 profiling work found correlations between 16-hour drug priming to BIM-BH3 and 72 hour apoptosis [32], so our much shorter, four-hour, culture is likely to be particularly relevant to agents with early pro-apoptotic effects.

In a clinical setting, the combination of an agent sensitising to BCL-2 antagonism and one sensitising to MCL-1 antagonism could be useful in a synthetic lethal combination, such as the combination of ABT-199 or a BET domain inhibitor with the FLT3 inhibitor AC220 documented in Figs 4 and 5. FLT3 internal tandem duplications affect 30% of AML patients [58]. The decision to use FLT3 inhibitors in this study was based on work showing the downregulation of MCL-1 to be a consequence of inhibition of translation initiation by sorafenib [26]. FLT3 internal tandem duplications drive constitutive activation of STAT5 and PI3K, both of which can drive MCL-1 overexpression through the translation initiation complex [59], such that MCL-1 downregulation is likely to be a common effect of diverse FLT3 inhibitors. Synergy between JQ1 and AC-220 has been reported [60]. The spliceosome inhibitor pladienolide B was also highly effective in combination with ABT-199 or JQ1 in the current study. Early evidence suggests that targeting of the spliceosome in cases of MDS or AML with spliceosome mutations could be effective [61].

The MV4-11 cells used in this study were sensitive to either BCL-2 or MCL-1 inhibition, and were used in order to focus on differences between drugs, rather than differences between cells. Cellular dependence on different members of the BCL-2 family is highly heterogeneous, even within a single disease such as AML, and possibly even within different clones from a single patient. Considerable further work will be needed to document the efficacy of co-operative drug combinations in cells with different BCl-2 family dependencies. In a previous report we showed that the complementarity between ABT-199 and TG02 was synergistic in some cases of AML, whereas in other cases we showed the combination to be effective when one or other of the agents alone was ineffective [18].

In conclusion we have used dynamic BH3 profiling to demonstrate that drugs sensitising to BCL-2 antagonism and drugs sensitising to MCL-1 antagonism can be systematically identified and to determine that dynamic co-operative BH3 profiling can predict drug combinations that induce rapid apoptosis.

## Supporting information

S1 Fig Sensitivity to ABT-199 and TW-37 in AML cell lines. We antagonised BCL-2 with ABT-199 [31] and MCL-1 with TW-37 [44]. The IC_50_s shown were obtained from alamar blue assays after treating 11 AML cell lines at a starting cell concentration of 2.5x10^5^ /ml for 48 hours. Each cell line thawed is tested around the time of its final passage to authenticate its provenance using the Powerplex 16 kit (Promega, Southampton, UK) to amplify short tandem repeats. The reactions are run on a 3130 Genetic Analyser and data analysed using Genemapper. Mycoplasma testing was carried out routinely using the Mycoalert mycoplasma detection kit (Lonza, Rockland, USA) and following the manufacturer’s instructions.
(TIF)

S2 Fig Dynamic BH3 profiling assay: Delta priming to PUMA-BH3 with additional anti-AML drugs. Delta priming is measured by cytochrome C release after 4 hour drug treatment and additional incubation with 3 μM PUMA-BH3. Values are corrected for Cytochrome C release with peptide only as described in the methods). (Mean+/- SD for n = 3). Suitable priming concentrations (>65% specificity) were established for the hsp90 inhibitor 17-AAG and the CRM1 inhibitor selinexor, but other agents were less effective. Hsp90 inhibitors [62] and selinexor [63] are reported to downregulate MCL-1. Tosedostat is reported to induce NOXA [64]. The contrast in priming abilities between rapamycin and torin1 (Fig 1) merits comment: this may be explicable in terms of the rapamycin insensitive effects of mTORC1 on 4E-BP1 [42]. 5-azacytidine (5-aza) and cytosine arabinoside (ara-C) were included for general interest.
(TIF)

S3 Fig Dynamic BH3 profiling assay: Delta priming with TG02 and ABT-199 to BAD-BH3 and MS1-BH3 peptides. Delta priming is measured by cytochrome C release after TG02 (50 nM) and ABT-199 (50 nM) treatment and additional incubation with the indicated BH3 peptides. Values are corrected for Cytochrome C release with peptide only as described in the methods). (Mean+/- SD for n = 3).
(TIF)

S4 Fig Dynamic BH3 profiling assay: Delta priming with additional anti-AML drugs to BAD-BH3 and MS1-BH3 peptides. Delta priming is measured by cytochrome C release after drug treatment and additional incubation with the indicated BH3 peptides (BAD-BH3 at 3 μM, MS1-BH3 at 3 μM, PUMA2A control at 100 μM). Values are corrected for Cytochrome C release with peptide only as described in the methods). (Mean+/- SD for n = 3).
(TIF)

S5 Fig Additional indicators of co-operative induction of apoptosis by ABT-199 with pladienolide B, torin1, etoposide and AC220: FACS plots. Cells were incubated with the indicated combinations of 10 nM ABT-199, 10 nM pladienolide B, 1 μM torin1, 1 μM etoposide or 10 nM AC220. After 4 hours cells were incubated for a further 75 minutes with DiOC_6_ to measure ↓Δψm. 7-AAD was added to the cells for the final 30 minutes of the incubation. The FACS plots illustrate that the treated cells stained by 7-AAD (indicating cell membrane permeability at a final stage of apoptosis) tend to lag very slightly behind cells with ↓Δψm, indicating rapid transition from ↓Δψm to irreversible apoptosis.
(TIF)

S6 Fig Dynamic BH3 profiling assay: A-1210477. Delta priming is measured by cytochrome C release after A-1210477 treatment and additional incubation with the indicated BH3 peptides. Values are corrected for cytochrome C release with peptide only as described in the methods. Results from priming with 10nm pladienolide are illustrated as positive control (<10% priming without peptide, strong priming with peptide) (Mean+/- SD for n = 3).
(TIF)
