# Supplementary figures and images for: Predicting effective pro-apoptotic anti-leukaemic drug combinations using co-operative dynamic BH3 profiling
Source: PLoS One. 2018 Jan 3;13(1):e0190682. doi: 10.1371/journal.pone.0190682 (PMC5752038; doi:10.1371/journal.pone.0190682)

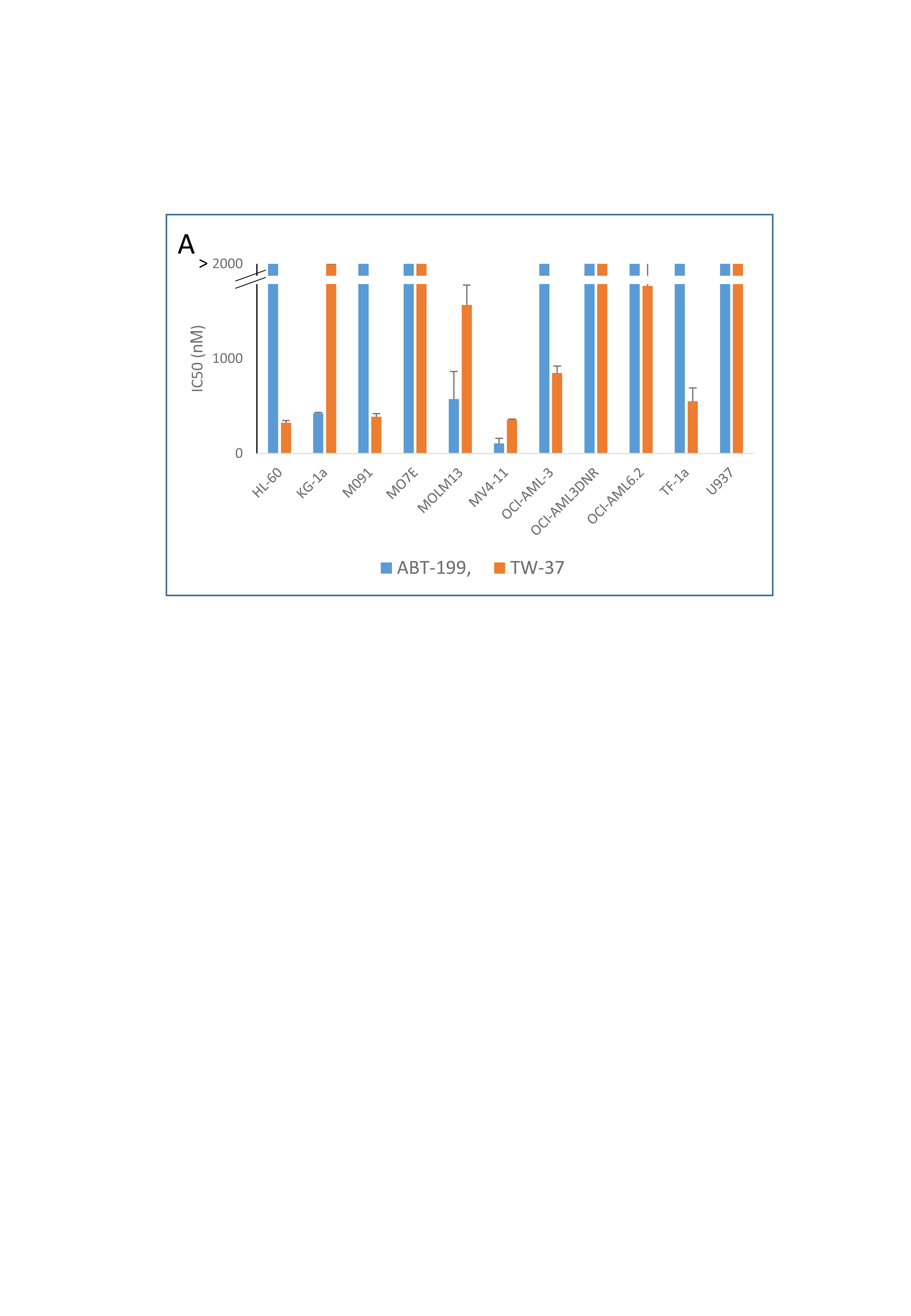

Supplement: S1 Fig — We antagonised BCL-2 with ABT-199 [31] and MCL-1 with TW-37 [44]. The IC50s shown were obtained from alamar blue assays after treating 11 AML cell lines at a starting cell concentration of 2.5x10^5^ /ml for 48 hours. Each cell line thawed is tested around the time of its final passage to authenticate its provenance using the Powerplex 16 kit (Promega, Southampton, UK) to amplify short tandem repeats. The reactions are run on a 3130 Genetic Analyser and data analysed using Genemapper. Mycoplasma testing was carried out routinely using the Mycoalert mycoplasma detection kit (Lonza, Rockland, USA) and following the manufacturer’s instructions. (TIF) [file pone.0190682.s001.tif]

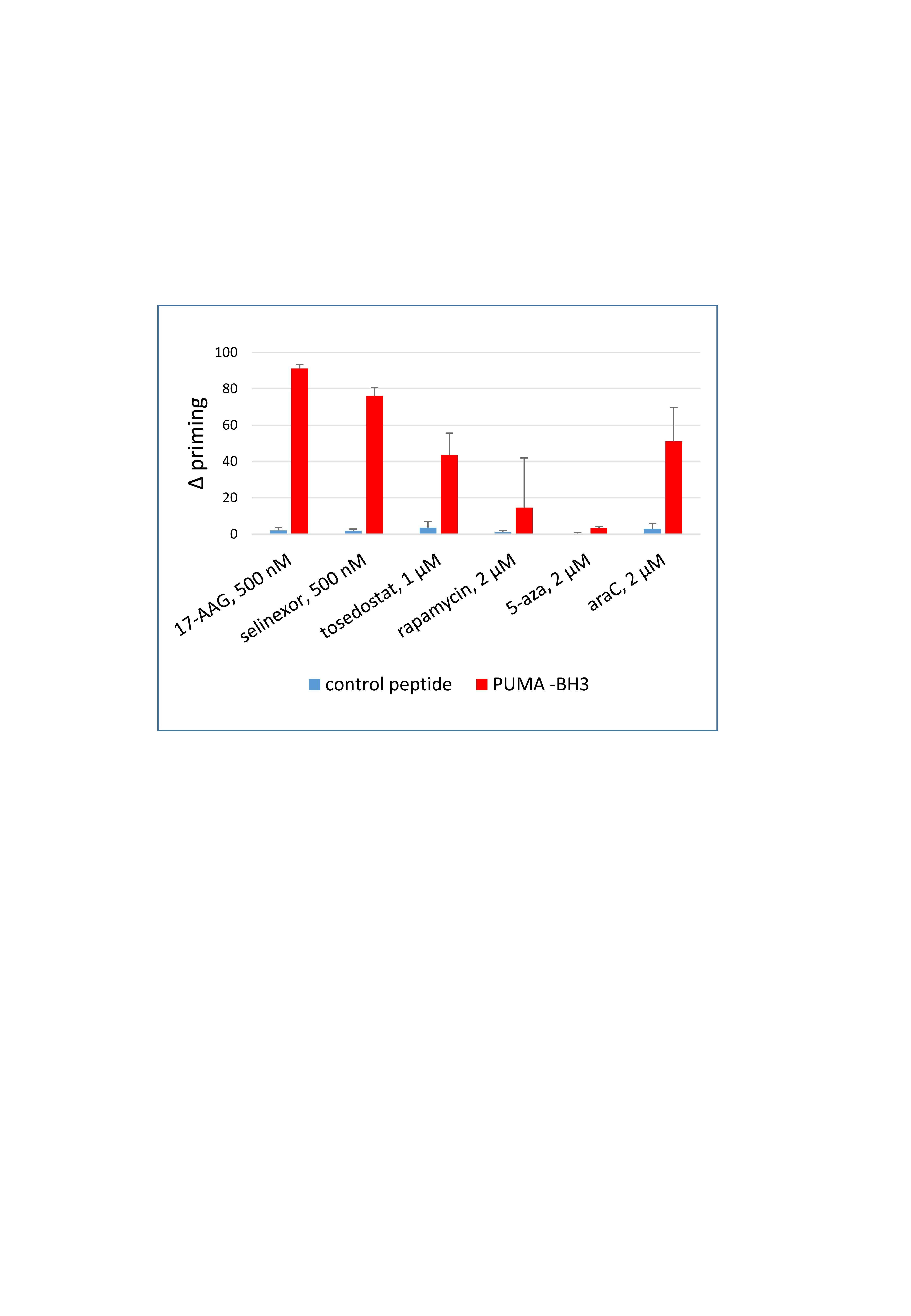

Supplement: S2 Fig — Delta priming is measured by cytochrome C release after 4 hour drug treatment and additional incubation with 3 μM PUMA-BH3. Values are corrected for Cytochrome C release with peptide only as described in the methods). (Mean+/- SD for n = 3). Suitable priming concentrations (>65% specificity) were established for the hsp90 inhibitor 17-AAG and the CRM1 inhibitor selinexor, but other agents were less effective. Hsp90 inhibitors [62] and selinexor [63] are reported to downregulate MCL-1. Tosedostat is reported to induce NOXA [64]. The contrast in priming abilities between rapamycin and torin1 (Fig 1) merits comment: this may be explicable in terms of the rapamycin insensitive effects of mTORC1 on 4E-BP1 [42]. 5-azacytidine (5-aza) and cytosine arabinoside (ara-C) were included for general interest. (TIF) [file pone.0190682.s002.tif]

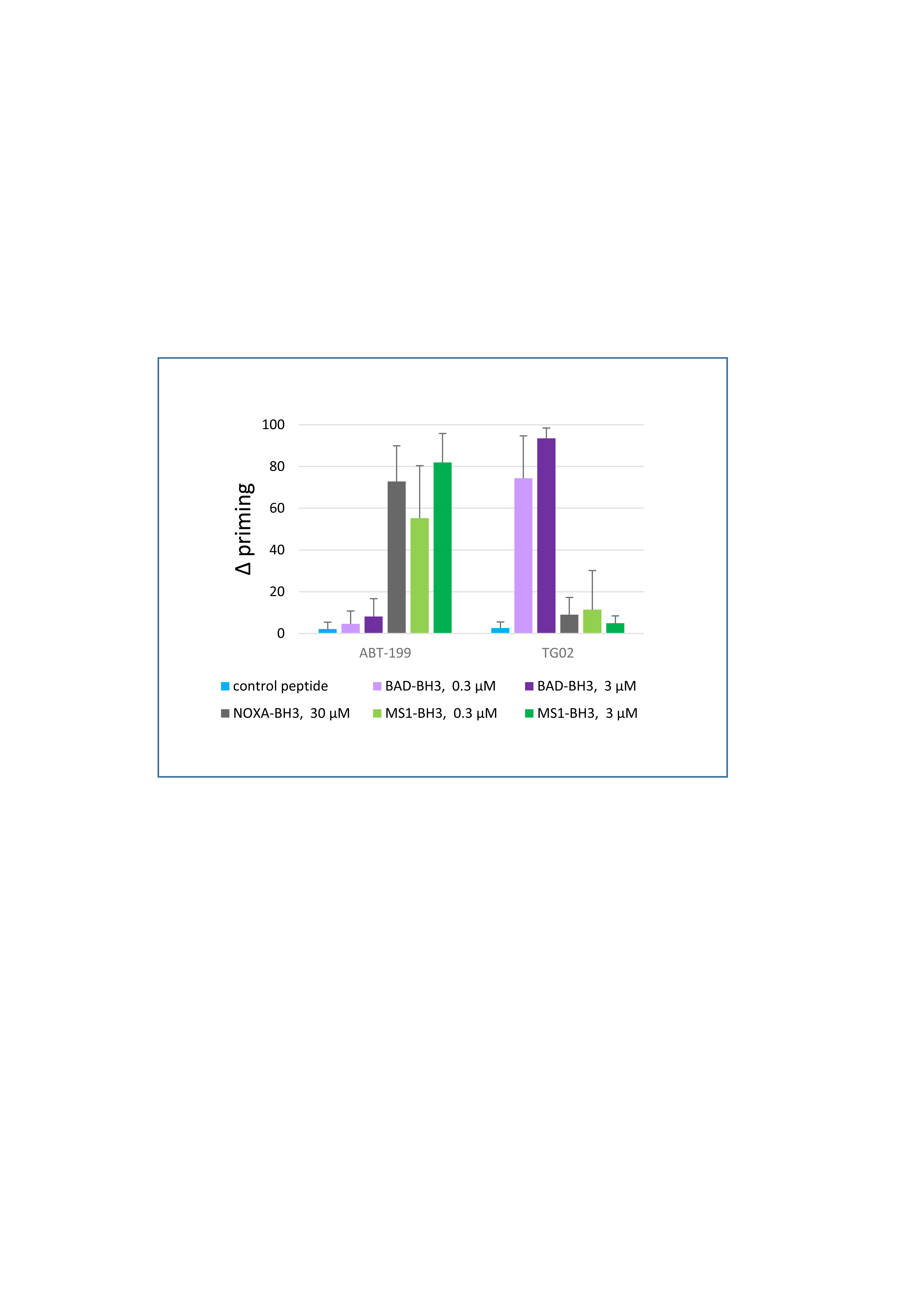

Supplement: S3 Fig — Delta priming is measured by cytochrome C release after TG02 (50 nM) and ABT-199 (50 nM) treatment and additional incubation with the indicated BH3 peptides. Values are corrected for Cytochrome C release with peptide only as described in the methods). (Mean+/- SD for n = 3). (TIF) [file pone.0190682.s003.tif]

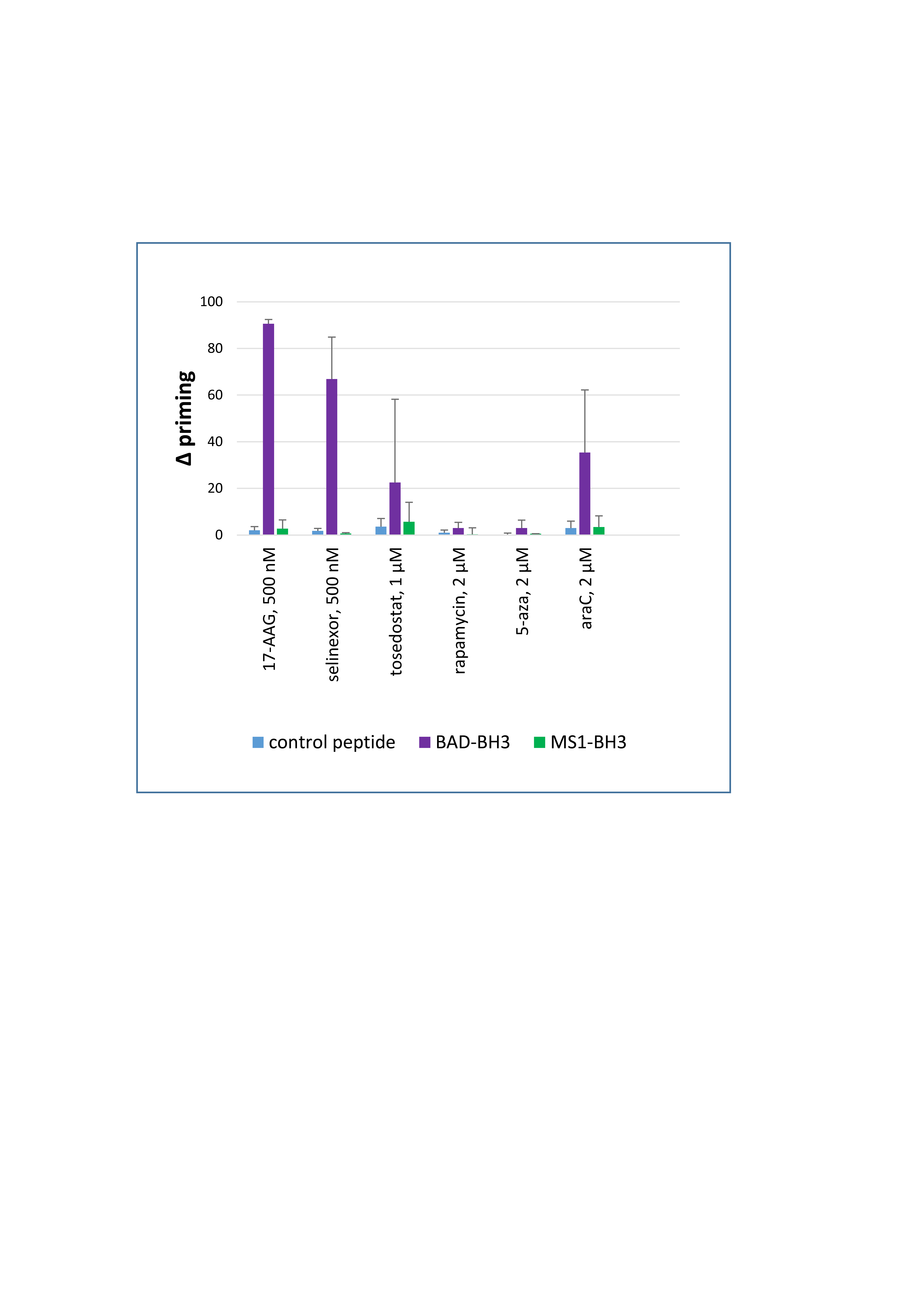

Supplement: S4 Fig — Delta priming is measured by cytochrome C release after drug treatment and additional incubation with the indicated BH3 peptides (BAD-BH3 at 3 μM, MS1-BH3 at 3 μM, PUMA2A control at 100 μM). Values are corrected for Cytochrome C release with peptide only as described in the methods). (Mean+/- SD for n = 3). (TIF) [file pone.0190682.s004.tif]

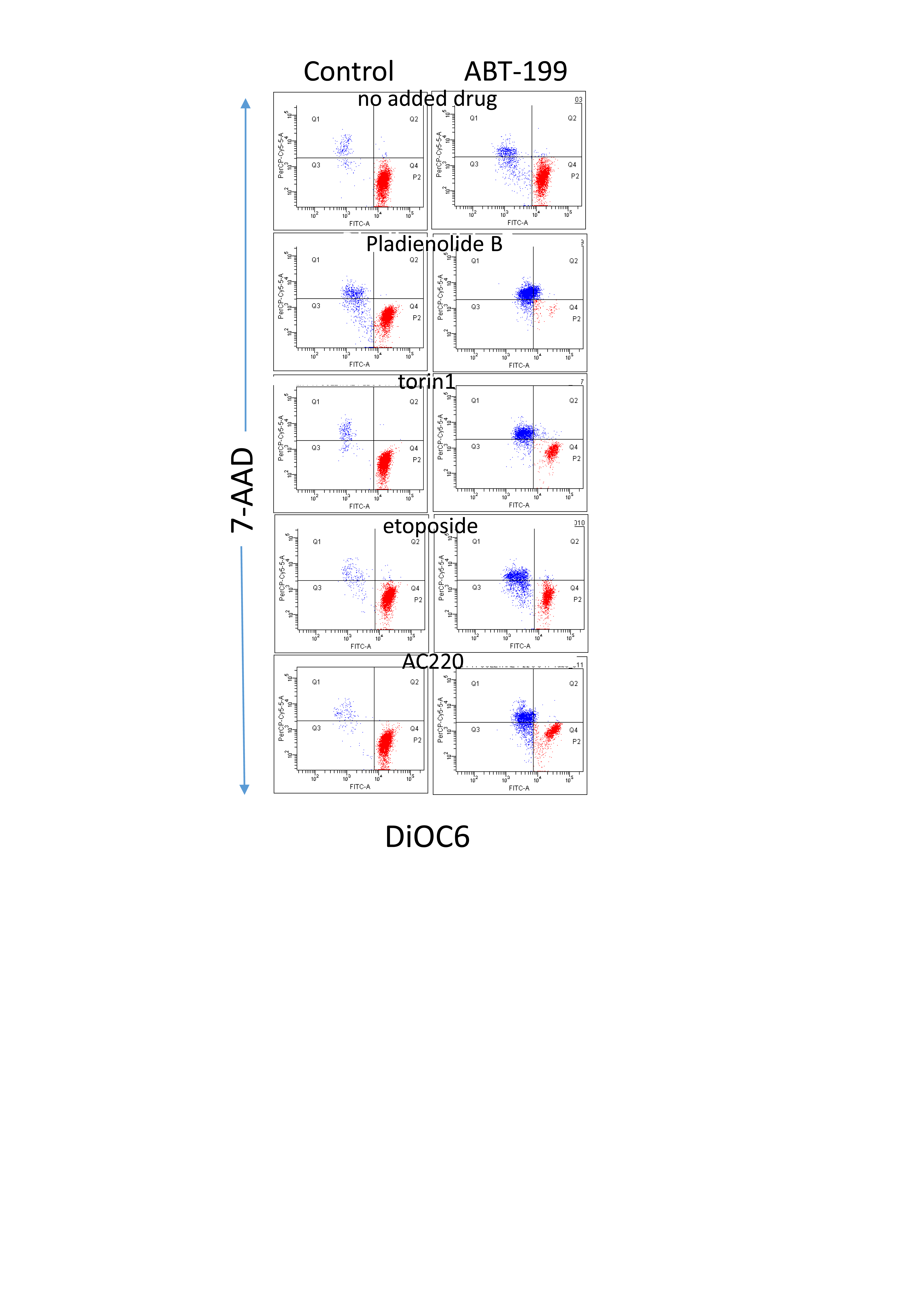

Supplement: S5 Fig — Cells were incubated with the indicated combinations of 10 nM ABT-199, 10 nM pladienolide B, 1 μM torin1, 1 μM etoposide or 10 nM AC220. After 4 hours cells were incubated for a further 75 minutes with DiOC6 to measure ↓Δψm. 7-AAD was added to the cells for the final 30 minutes of the incubation. The FACS plots illustrate that the treated cells stained by 7-AAD (indicating cell membrane permeability at a final stage of apoptosis) tend to lag very slightly behind cells with ↓Δψm, indicating rapid transition from ↓Δψm to irreversible apoptosis. (TIF) [file pone.0190682.s005.tif]

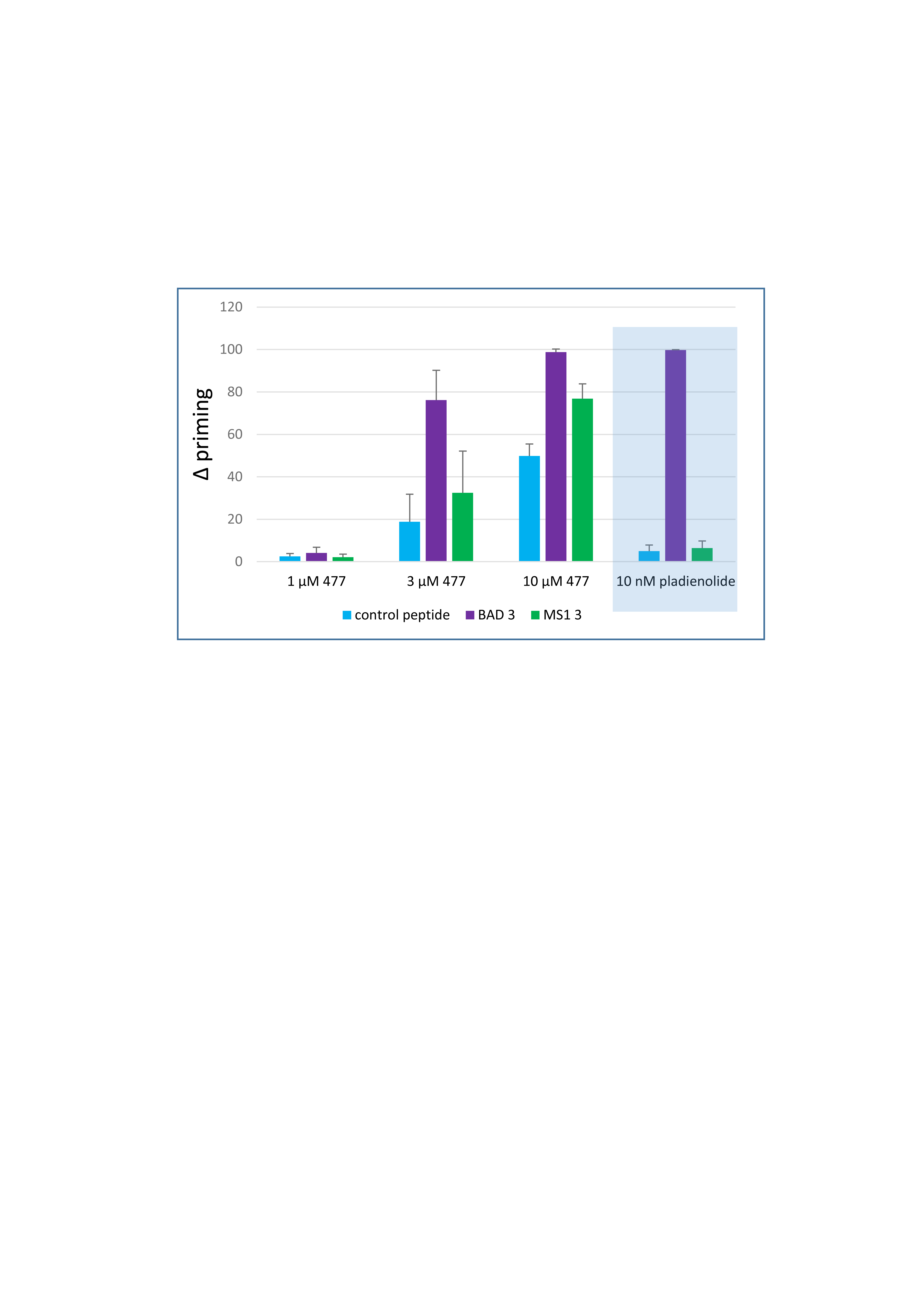

Supplement: S6 Fig — Delta priming is measured by cytochrome C release after A-1210477 treatment and additional incubation with the indicated BH3 peptides. Values are corrected for cytochrome C release with peptide only as described in the methods. Results from priming with 10nm pladienolide are illustrated as positive control (<10% priming without peptide, strong priming with peptide) (Mean+/- SD for n = 3). (TIF) [file pone.0190682.s006.tif]
